# Comprehensive review of adverse reactions and toxicology in ASO-based therapies for Duchenne Muscular Dystrophy: From FDA-approved drugs to peptide-conjugated ASO

**DOI:** 10.1016/j.crtox.2024.100182

**Published:** 2024-06-18

**Authors:** Umme Sabrina Haque, Melissa Kohut, Toshifumi Yokota

**Affiliations:** a Department of Neuroscience, Faculty of Medicine and Dentistry, University of Alberta, Edmonton, AB T6G 2H7, Canada; b Department of Medical Genetics, Faculty of Medicine and Dentistry, University of Alberta, Edmonton, AB T6G 2H7, Canada; c The Friends of Garrett Cumming Research & Muscular Dystrophy Canada HM Toupin Neurological Science Research, Edmonton, AB T6G 2H7, Canada

**Keywords:** DMD, Eteplirsen, Golodirsen, Viltolarsen, Casimersen, SRP-5051, PGN-ED051, ENTR-601-44, Adverse rection, Toxicity

## Abstract

Duchenne Muscular Dystrophy (DMD) is a devastating X-linked genetic disorder characterized by progressive muscle degeneration due to mutations in the dystrophin gene. This results in the absence or dysfunction of the dystrophin protein, leading to muscle weakness, loss of ambulation, respiratory issues, and cardiac complications, often leading to premature death. Recently, antisense oligonucleotide (ASO)-mediated exon skipping has emerged as a promising therapeutic strategy for DMD. Notably, the FDA has conditionally approved four ASO therapies for DMD, with numerous others in various stages of clinical development, indicating the growing interest and potential in this field. To enhance ASO-based therapies, researchers have explored the novel concept of conjugating peptides to the phosphorodiamidate morpholino backbone (PMO) of ASOs, leading to the development of peptide-conjugated PMOs (PPMOs). These PPMOs have demonstrated significantly improved pharmacokinetic profiles, potentially augmenting their therapeutic effectiveness. Despite the optimism surrounding ASOs and PPMOs, concerns persist regarding their efficacy and safety. To comprehensively evaluate these therapies, it is imperative to expand patient populations in clinical trials and conduct thorough investigations into the associated risks. This article provides a comprehensive review and discussion of the available data pertaining to adverse reactions and toxicology associated with FDA-approved ASO drugs for DMD. Furthermore, it offers insights into the emerging category of peptide-conjugated ASO drugs those are clinical and preclinical trials, shedding light on their potential benefits and challenges.

## Highlights

• DMD is a devastating genetic disease, causing muscle breakdown and premature death.
• ASOs offer hope, with four FDA-approved therapies and growing interest.
• Peptide conjugation can enhance the pharmacokinetic profiles of ASO-based therapies.
• Clinical trials with ASOs and peptide-conjugated ASOs have revealed adverse effects.
• Larger clinical trials and risk assessment is crucial to evaluate these therapies.

## Graphical abstract

**Figure:**
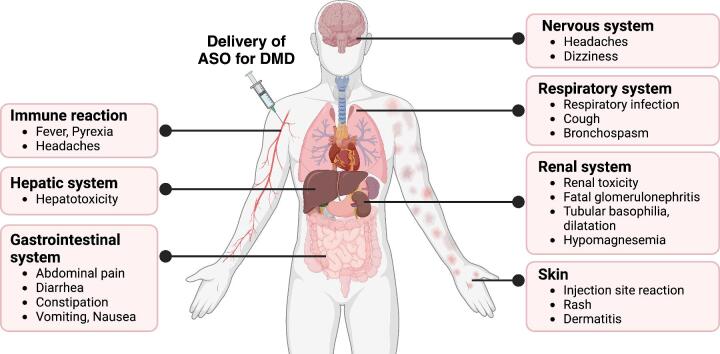

## 1 Introduction

The underlying pathology of DMD arises from mutations in the dystrophin (*DMD*) gene, resulting in the absence of dystrophin protein(Hoffman et al., 1987, Duan et al., 2021). This critical protein is expressed in various tissues, including the heart, skeletal muscles, smooth muscles, retina, and brain (Duan et al., 2021, Deisch, 2017). Dystrophin plays a pivotal role in maintaining muscle stability by forming a scaffold that connects actin filaments to the sarcolemma. In the absence of dystrophin, muscle fibers are susceptible to progressive degradation and fibrosis (Gao and McNally, 2015).

The onset of DMD symptoms is typically evident in early childhood (2–5 years), characterized by delayed walking ability, a waddling gait, impaired movements, and a tendency to fall frequently (Flanigan, 2014, Henricson et al., 2013). As the disorder advances, muscle weakness and joint contractures intensify, leading to most patients becoming wheelchair-dependent by the ages of 10–12 (Yiu and Kornberg, 2015). The impact extends further into adolescence, with patients, between 18 and 20 years of age, often requiring ventilatory support as the disease affects respiratory and cardiac muscles (Lo Cascio et al., 2014, Kohler et al., 2005, Mavrogeni et al., 2015, Fayssoil et al., 2017). Beyond its physical manifestations, DMD exhibits a cognitive impairment component characterized by deficits in short-term memory, multitasking, procedural learning, and problem-solving. This cognitive aspect is attributed to dysfunctions in the cerebro-cerebellar pathway and has become a focal point for in-depth exploration (Rae and O'Malley, 2016, Battini et al., 2018, Vicari et al., 2018). Tragically, the progression of DMD proves fatal for most patients, with DMD-related cardiomyopathy emerging as the primary cause of death and a median lifespan of only 28 years (Broomfield et al., 2021).

*DMD*, the largest known human gene, is located in a genomic region with high rates of recombination (Ahn and Kunkel, 1993, Roberts et al., 1993). Due to its length and location, DMD is associated with over 4,000 mutations, primarily involving exon deletions, exon duplications, and small mutations like point mutations. The majority of these mutations induce a shift in the open reading frame, leading to the generation of premature stop codons and the exclusion of one or more exons. This results in the production of dystrophin protein is apparently not functional and therefore unstable (Aartsma-Rus et al., 2006, Tuffery-Giraud et al., 2009, Kyrychenko et al., 2017). Mutations in the *DMD* gene tend to cluster in two main hot-spot regions. One hot spot is located within the 5′ region of the gene, encompassing exons 2–20. These mutations constitute around 15 % of all exon deletions and about 50 % of all exon duplications within the DMD gene. Notably, deletions of exons 3–7 are the most prevalent in this region. The second significant DMD hot spot is situated in the distal region of the gene, covering exons 45–55, contributing to roughly 70 % of all exon deletions and approximately 15 % of all exon duplications (Bladen et al., 2015, Yang et al., 2013).

The dystrophin protein, comprising 3,685 amino acids, can be segmented into four distinct domains: (i) The actin-binding domain 1 (ABD-1), spanning amino acids 14–240, serves to link the filamentous elements of the cytoskeleton to the cell membrane, (ii) The central rod domain, characterized by 24 spectrin-like repeats and including the second actin-binding domain (ABD-2), (iii) The cysteine-rich domain, (iv) The carboxyl-terminal domain. Collectively, these domains enable dystrophin to function as a structural bridge between the cytoskeleton and extracellular matrix, crucial for maintaining muscle integrity. Dystrophin exhibits a degree of tolerance to internal deletions, as long as a subset of rod domains with intact amino- and carboxyl-termini regions is preserved, resulting in a mild loss of muscle function. Removal of a specific exon or exons can contribute to the stabilization of the DMD transcript by eliminating a mutant stop codon within the exon or by reinstating the reading frame of the downstream protein-coding sequence. Internal in-frame deletions in DMD occur naturally, giving rise to populations with milder allelic forms of muscular dystrophy. To mitigate the severity of DMD, diverse strategies have been employed, such as selectively skipping or deleting out-of-frame exons, thereby facilitating the expression of truncated forms of the dystrophin protein (Kyrychenko et al., 2017).

One such strategy is Antisense Oligonucleotide (ASO) based therapy. ASOs, consisted of short synthetic nucleic acid sequences, bind to complementary target pre-mRNA sequences, thereby influencing mRNA expression through diverse mechanisms such as splice switching, gene knockdown, gene upregulation, or modification of polyadenylation (Roberts et al., 2020, Aartsma-Rus and van Ommen, 2007, Lim et al., 2020, Khorkova et al., 2013, Marsollier et al., 2018). Regarding the treatment of Duchenne Muscular Dystrophy (DMD), the majority of current antisense therapies employ the splice-switching approach. This method aims to selectively skip *DMD* exons by binding adjacent to the deleted exon, thereby restoring the reading frame of dystrophin (Aartsma-Rus and van Ommen, 2007). Notably, four antisense oligonucleotide (ASO) candidates have received conditional FDA approval for DMD treatment since 2016, and numerous others are currently undergoing clinical and preclinical trials (Shirley, 2021, Lim et al., 2017, Roshmi and Yokota, 2019, Anwar and Yokota, 2020).

All four FDA-approved drugs for Duchenne Muscular Dystrophy (DMD) fall within the category of phosphorodiamidate morpholino oligomers (PMOs), wherein the five-membered ribose heterocycle is substituted by a six-membered morpholine ring structure with phosphorodiamidate linkages. PMOs have emerged as preferred agents for exon skipping in DMD therapy due to their superior attributes compared to Locked Nucleic Acid (LNA) or Morpholino Oligonucleotide with a 2′-O-methyl RNA backbone (MOE). PMOs, being neutrally charged, exert their antisense effect through steric hindrance or splice modulation (Hudziak et al., 1996). Their lack of a carbonyl group renders them resistant to degradation by proteases and nucleases, while their neutral charge reduces the likelihood of provoking immune responses (Lee and Yokota, 2013). Additionally, studies have indicated that multiple high doses of PMOs can be administered with minimal toxicity(Sheng et al., 2020).

Despite being a promising approach, there are certain limiting factors of ASO as a therapeutic agent such as challenges in intracellular trafficking, degradation in biological fluids and less distribution to required tissues (Shadid et al., 2021). Once systemically administered they need to be nuclease resistant, avoid renal clearance and the reticuloendothelial system (ALLEN, 1994, TSUI et al., 2002, IVERSEN et al., 2009, GEARY et al., 2015). Furthermore, due to the short duration of the exon skipping effect ASOs often require high drug dosages (Haque and Yokota, 2023).

To address these concerns, attempts have been made to conjugate ASOs with cell-penetrating peptides (CPPs). These small peptides have the ability to transport cargos, including ASOs, across cellular barriers, potentially improving cellular uptake, intracellular distribution, and therapeutic outcomes while reducing the required dosage (Tsoumpra et al., 2019, Moulton and Moulton, 2010). However, several studies have reported several concerns including toxicity associated with peptide-conjugated ASOs along with FDA-approved ASO. Current CPP that are being investigated are rich in arginine and 6-aminohexanoic acid residues which allows for increased cell infiltration. Studies have shown that the infiltration ability of the peptides correlates with the numbers of arginine and/or 6-aminohexanoic acid residues (Wu et al., 2007). However, the increased amount of 6-aminohexanoic acid residues increases cell death in vitro (Wu et al., 2007). Furthermore, these CPP rich in arginine and 6-aminohexanoic acid residues have shown renal toxicity in rodent models (Amantana et al., 2007). Some studies have used D-arginine residues instead of L-arginine residues which has shown greater resistance to proteases and reduced cytotoxicity but may decrease cellular uptake efficiency in some cell types (WU et al., 2007, PUJALS and GIRALT, 2008, VERDURMEN et al., 2011). Therefore, in order to have a CPP with enhanced cellular uptake and low toxicity, the number and order of arginine residues and 6-aminohexanoic acid residues requires optimization.

This article aims to provide an update on the adverse reaction and toxicology of FDA-approved Antisense Oligonucleotide (ASO) and peptide-conjugated ASO (in clinical or preclinical trial), incorporating findings from pre-clinical and clinical trials and addressing issues in their path to definitive approval.

## 2 Adverse reaction and toxicity of FDA approved antisense oligonucleotide based drug for DMD

As of the current writing, the FDA has conditionally approved four Antisense Oligonucleotides (ASOs) targeting three distinct exons for Duchenne Muscular Dystrophy (DMD) treatment: eteplirsen (Sarepta, exon 51 skipping) (FDA, 2016), golodirsen (Sarepta, exon 53) (FDA, 2019), viltolarsen (NS pharma, exon 53) (FDA, 2020a) and casimersen (Sarepta, exon 45) (FDA, 2021b). All four drugs belong to the phosphorodiamidate morpholino oligomer (PMO) class of synthetic ASOs. PMOs are characterized by the substitution of the deoxyribose moiety with a morpholine ring, and the charged phosphodiester inter-subunit linkage is replaced by a non-ionic phosphorodiamidate linkage. This chemical structure imparts a high affinity for target nucleic acid sequences and renders PMOs less susceptible to nuclease activity compared to conventional nucleic acids like DNA and RNA. Furthermore, PMOs operate by interfering with the binding and interaction of splice nucleic acids and proteins, preventing translation rather than reducing transcript levels as seen in RNase H-mediated breakdown(Summerton and Weller, 1997, Hudziak et al., 1996). While all four candidates have completed initial phase III clinical trials successfully, these drugs raise several concerns, including limited dystrophin protein production and potential toxic effects (Table 1).

**Table 1:** Adverse reaction, toxicity and warning of the FDA-approved PMO based ASO drugs and peptide conjugated PMO in clinical/preclinical trial/Phase I Clinical Trials.

Name	Sponsor	Therapeutic target	ASO chemistry	Status	Formulation	Dosing	Adverse drug reaction	FDA warnings
Eteplirsen (Exondys 51)	Sarepta Therapeutics	Exon 51 Skipping	PMO	Conditionally Approved (2016)	30 mg/kg	35–60 min infusion once weekly	Lower respiratory infection, pyrexia, constipation, headache, vomiting, and hypersensitivity reaction	Hypersensitive reactions
Golodirsen (Vyondys 53)	Sarepta Therapeutics	Exon 53 Skipping	PMO	Conditionally Approved (2019)	30 mg/kg	35–60 min infusion once weekly	Headaches, pyrexia, fall, abdominal pain, nasopharyngitis, cough, vomiting, and nausea. Renal toxicity (according to preclinical data)	Renal toxicity, Hypersensitive reactions
Viltolarsen (Viltepso)	NS Pharma	Exon 53 Skipping	PMO	Conditionally Approved (2020)	80 mg/kg	60 min infusion once weekly	Upper respiratory tract infection, Injection site reaction, cough, and pyrexia.	Renal toxicity
Casimersen (Amondys 45)	Sarepta Therapeutics	Exon 45 Skipping	PMO	Conditionally Approved (2021)	45–30 mg/kg	35–60 min infusion once weekly	Upper respiratory tract infections, cough, pyrexia, headache, arthralgia, and oropharyngeal pain	Renal toxicity
SRP-5051	Sarepta Therapeutics	Exon 51 Skipping	PPMO	Phase II	N/A	N/A	Hypomagnesemia	N/A
PGN-ED051	PepGen	Exon 51 Skipping	PPMO	Phase I	N/A	N/A	Kidney toxicity, hypomagnesemia	N/A
ENTR-601-44	Entrada Therapeutics	Exon 44 Skipping	PPMO	Preclinical	N/A	N/A	N/A	N/A

The information for formulation and dosing is extracted from the FDA drug labels (FDA, 2024).

### 2.1 Eterlipsen

Eteplirsen is the first ASO-based drug for Duchenne muscular dystrophy (DMD) that got FDA approval on September 19, 2016 (FDA, 2016). The accelerated approval was mainly granted based on the observed changes in dystrophin expression levels. In biopsies taken from 11 patients after 180 weeks of treatment, there was a notable increase of 0.93 % compared to the initial baseline value of 0.08 %. Similarly, biopsies from 13 patients after 48 weeks of treatment showed a lesser but still significant increase of 0.28 % compared to baseline (Aartsma-Rus and Goemans, 2019). Despite showing promising signs it has raised concerns including limited dystrophin production, uncertainty about its effectiveness in exon-skipping, and variations in efficacy linked to the DMD mutation pattern (Kesselheim and Avorn, 2016, Aartsma-Rus and Goemans, 2019). Additionally, in a phase 2 clinical study with 12 participants (NCT01396239), adverse drug reactions (ADRs) were reported at least 25 % higher than placebo (Mendell et al., 2013). This trial was a double-blind, placebo-controlled, randomized study where patients received weekly intravenous infusions of 30 and 50 mg/kg eteplirsen or placebo for 24 weeks. Phase II trials reported ADRs like balance disorder, vomiting, and contact dermatitis, with higher rates than the placebo group(Mendell et al., 2013). In a subsequent phase 3 clinical trial (NCT02255552) with 109 patients receiving at least 30 mg/kg/week of eteplirsen for up to 144 weeks, some adverse events were observed. These events included vomiting, contusion, excoriation, arthralgia, rash, catheter site pain, and respiratory tract infection, with at least 10 % of patients reporting these symptoms. Less than 1 % of patients experienced bronchospasm, cough, dyspnea, fever, flushing, hypersensitivity reactions, hypotension, and urticaria, as reported in postmarketing surveillance and case reports. It is important to note that in clinical trials, some patients treated with eteplirsen exhibited hypersensitivity reactions, including rash, pyrexia, flushing, urticaria, bronchospasm, dyspnea, and cough (Alhamadani et al., 2022). Therefore, the FDA label highlights the possibility of hypersensitivity reactions associated with this therapy. No adverse events led to treatment interruption, dose changes, discontinuation, or deaths. In some eteplirsen recipients, protein was detected in 19 out of 609 urine samples over about three years, but levels were generally low, transient, and resolved without renal toxicity. Eteplirsen showed no hepatic toxicity, and in fact, it led to decreases in creatine kinase, aspartate aminotransferase, and alanine aminotransferase levels associated with DMD over treatment duration (Mendell et al., 2016). Apart from that, since the approval of eteplirsen in 2016, a retrospective comparative analysis has shown that this treatment has reduced the burden of this disease most significantly by decreasing hospital (31 %) and emergency admissions and reducing the requirement for pulmonary management (33 %), cardiac management (21 %), tracheostomies (86 %), and assisted ventilation (39 %) compared to those receiving standard of care (Iff et al., 2023).

### 2.2 Golodirsen

Golodirsen, the second FDA-approved drug for Duchenne muscular dystrophy (DMD), gained approval on 12 December 2019 based on increased dystrophin expression (FDA, 2019). An increase of 0.924 % of dystrophin from baseline was observed after 48 weeks of golodirsen treatment (Frank et al., 2020). The adverse reaction of this drug was reported in a phase 1/2 clinical trial (NCT02310906), which consisted of a randomized, placebo-controlled dose-titration study (part 1) followed by an open-label evaluation (part 2) involving 39 participants(Frank et al., 2020). This trial reported common adverse reactions occurring in at least 20 % of patients including headaches, pyrexia, fall, abdominal pain, nasopharyngitis, cough, vomiting, and nausea. Some patients treated with golodirsen did show non-serious signs of hypersensitivity reactions (Servais et al., 2022). 1 participant (4 %) had a rash and 3 participants (12 %) showed pyrexia. An extension study (NCT03532542) is currently enrolling participants for a phase 3 study with an estimated 260 participants, expected to conclude in August 2026, to further assess golodirsen in patients with Duchenne muscular dystrophy (DMD)(Alhamadani et al., 2022). Although renal toxicity was not observed in clinical trials of golodirsen, preclinical studies in animals (adult male C57BL/6NCrl mouse and cynomolgus monkey and in male juvenile Sprague Dawley rat) revealed kidney problems, including tubular degeneration and dysfunction. Mice and primates exhibited kidney dysfunction, with accompanying changes such as tubular basophilia, dilatation, and mononuclear cell infiltration (Bilal Abuasal et al., 2019). Considering animal studies that have suggested the potential for golodirsen to induce renal toxicity, it is advisable to closely monitor renal function in patients undergoing golodirsen treatment. This monitoring should encompass the assessment of glomerular filtration rate (GFR) before starting treatment and the ongoing surveillance for any signs of renal toxicity during the course of treatment (Sarepta Therapeutics, 2019). Nevertheless, it is worth noting that creatinine clearance is not deemed a dependable metric for characterizing renal function in the DMD population due to the predominant impact of disease on muscles (Viollet et al., 2009, Screever et al., 2021). Additionally, hypersensitivity reactions, characterized by symptoms like rash, pruritus, pyrexia, dermatitis, urticaria, and skin exfoliation, were observed in clinical trials with golodirsen-treated patients. Therefore, the FDA-approved label for golodirsen includes warnings about hypersensitivity reactions and emphasizes concerns regarding potential renal toxicity(Sarepta Therapeutics, 2019). As the pivotal trials of golodirsen had limited patient numbers, the complete hepatotoxicity profile is unclear. However, these trials didn't show liver issues like elevated aminotransferase or discontinuations due to liver problems, indicating no link to acute hepatitis or jaundice (Bilal Abuasal et al., 2019).

### 2.3 Viltolarsen

Viltolarsen gained accelerated FDA approval on 12 July 2020 for its ability to increase dystrophin levels in the skeletal muscles of DMD patients (FDA, 2020a). In a phase 2 dose-finding clinical study, the mean increase in dystrophin production was found to be 5.7 % and 5.9 % from baseline in 40 and 80 mg/kg dose cohorts, respectively (Clemens et al., 2020). Viltolarsen was generally well tolerated in the phase 2 study (NCT02740972) involving 16 participants with mild or moderate reactions after 24 weeks’ of viltolarsen administration at a dosage of 40 or 80 mg/kg once weekly. Adverse reactions were observed in a phase 2 clinical trial (NCT02740972) involving 16 participants(Clemens et al., 2020). Common adverse reactions, similar to golodirsen, included injection site reactions, upper respiratory tract infections, cough, and pyrexia (observed in >15 % of treated patients), while less common adverse reaction encompassed contusion, arthralgia, diarrhea, vomiting, and abdominal pain(Clemens et al., 2020). In a Japanese study (JapicCTI-163291), viltolarsen at 40 or 80 mg/kg once weekly was well-tolerated in a similar patient population. All treatment-related adverse events were mild or moderate, with none leading to treatment discontinuation. In the 80 mg/kg group (8 patients), common adverse events included nasopharyngitis, upper respiratory tract infection (3 patients each), and pyrexia, urticaria, increased β-N-acetyl-D-glucosaminidase, and decreased ejection fraction (2 patients each)(Komaki et al., 2018). At the 40 mg/kg dose, no urticaria or pyrexia were observed suggesting no hypersensitivity reactions (Komaki et al., 2020). However, 2 participants (25 %) exhibited urticaria or pyrexia indicating the potential to cause hypersensitivity reactions at the higher dose. There is no hypersensitivity warning and precaution for viltolarsen from FDA. Preclinical studies in animal model (male CD-1 mouse (13 and 26 weeks) and male cynomolgus monkey (12 and 39 weeks)) revealed renal toxicity at higher doses of viltolarsen (Freed, 2020). Mice receiving 240 and 1,200 mg/kg doses exhibited a dose-dependent increase in renal toxicity-related deaths and severity of renal tubular effects, including degeneration. The highest dose also led to reduced body weight and delayed sexual maturation. However, at a dose of 60 mg/kg, no renal toxicity was observed, and plasma exposure was comparable to that in humans receiving 80 mg/kg (Freed, 2020). Long-term animal studies for assessing carcinogenic potential were not conducted, and no adverse effects on embryofetal development were found in animal studies. Although clinical trials with limited patient samples did not initially detect renal toxicity, the FDA has issued warnings regarding the potential risk of renal toxicity and fatal glomerulonephritis associated with viltolarsen (FDA, 2020b). Viltolarsen therapy has shown no evidence of liver injury, including no notable serum enzyme elevations or clinically apparent liver problems. There were no treatment discontinuations due to adverse effects (Clemens et al., 2022). Postmarket data for viltolarsen is currently unavailable given the limited time since its approval.

### 2.4 Casimersen

Casimersen is the latest FDA-approved ASO drug for DMD which got approval on 25 February 2021 (FDA, 2021b). A mean dystrophin change of 0.81 % was seen 48 weeks following the start of casimersen therapy in a placebo-controlled clinical trial Iannaccone, 2022). In its phase 1 clinical trial with 12 participants (NCT02530905), common adverse reactions (>20 % of patients) included upper respiratory tract infections, cough, pyrexia, headache, arthralgia, and oropharyngeal pain (Wagner et al., 2021). The trial involved males aged 7–21 with specific DMD genetic mutations. Patients were randomized to receive escalating doses of casimersen at 4, 10, 20, and 30 mg/kg or placebo once a week for 12 weeks, followed by open-label casimersen treatment at 30 mg/kg for up to 132 weeks (Wagner et al., 2021). There is an ongoing extension study (ClinicalTrial.gov ID: NCT03532542) with 260 participants expected to conclude in 2026 and another multicenter study called ESSENCE with 222 participants set to finish in 2024. Preclinical studies revealed kidney toxicity in both adult male C57BL/6NCrl mice and cynomolgus monkeys (Freed, 2021). However, kidney toxicity was not observed in the limited phase 1/2 clinical study (12 participants for both treatment and control) (Wagner et al., 2021). Despite this, the FDA includes warnings and precautions for kidney toxicity in the drug labeling of casimersen (FDA, 2021a), similar to golodirsen and viltolarsen. The kidney toxicity warning of FDA is not based on signs of kidney issues in clinical trials but reflects the risk associated with the ASO class of medications as a whole. Given the recent FDA approval of casimersen and its limited patient population, there is currently a lack of post-marketing safety surveillance data. More data and information are needed to better understand its overall safety profile.

### 2.5 Alternative approaches

**scAAV9.U7.ACCA (in phase-1/2 clinical trials):** All the currently approved ASO-based drugs for Duchenne muscular dystrophy (DMD) target mutations in the exon 43–55 hotspot, leaving patients with mutations in the N-terminal-encoding exon 2–22 hotspot without treatment options. Kevin Flanigan and team from Nationwide Children’s Hospital have developed a gene therapy, scAAV9.U7.ACCA, which is undergoing phase-1/2 clinical trials to treat DMD patients with a duplication of exon 2. Unlike currently approved therapies that use exon-skipping therapy, Astellas' approach utilizes modified non-coding U7 small nuclear RNA (U7snRNA) molecules to induce exon skipping of one of the exon 2 duplicates, aiming to restore regular full-length dystrophin production. These snRNAs are designed to bind to splice acceptor and donor sites flanking exon 2, preventing other splicing enzymes from binding and causing the exclusion of one of the exon 2 duplicates from the final mRNA transcript (Wein et al., 2022). This exclusion aims to restore the production of regular full-length dystrophin, a protein crucial for muscle function. Preclinical studies in mice have shown promising results, demonstrating exon skipping and amelioration of the DMD phenotype. Additionally, safety studies conducted in mice and nonhuman primates have indicated low off-target activity and no significant toxicity at varying doses of scAAV9.U7.ACCA. Moving into clinical trials, a phase-I/II trial (NCT04240314) has been initiated to evaluate the safety and preliminary efficacy of scAAV9.U7.ACCA in DMD patients with confirmed exon 2 duplication. The trial, an open-label study, involves administering the therapy via peripheral limb vein injection to eligible male participants aged 6 months to 14 years. Over a period of two years, participants will be closely monitored for adverse effects, with dystrophin expression assessed through muscle biopsies and exon 2 exclusion quantified via RT-PCR. The outcomes of this trial will provide critical insights into the safety and efficacy of scAAV9.U7.ACCA, potentially informing future clinical developments in the treatment of DMD. The trial is scheduled to conclude by November 2025 (Wilton-Clark and Yokota, 2022).

**WVE-N531:** WVE-N531, developed by Wave life sciences, is an exon skipping therapy utilizing their phosphoryl guanidine-containing (PN) backbone chemistry modifications for individuals with DMD amenable to exon 53 skipping. Currently undergoing a Phase 1b/2a open-label study named FORWARD-53, this drug aims to evaluate the safety, tolerability, pharmacokinetic (PK), pharmacodynamic (PD), and clinical effects of intravenous (IV) WVE-N531 in DMD patients (NCT04906460).Phase 1b/2a Part A proof-of-concept trial in boys with DMD amenable to exon 53 skipping showed promising results, with all treatment-related adverse events being mild and the treatment appearing safe and well-tolerated (GlobeNewswire, 2022).

**NS-089/NCNP-02:** NS-089/NCNP-02, an exon skipping for those amenable to exon 44 skipping, developed by NS Pharma. NS Pharma has initiated an exploratory clinical trial in Japan in 2019 where they found this drug to be well-tolerated after infusion of 1.62, 10, 40, and 80 mg/kg in 6 DMD boys for 24 weeks with a 10.27 % (for 40 mg/kg) and 15.79 % (for 80 mg/kg) increase in dystrophin level. NS-089/NCNP-02 is currently in a phase II extension study (NCT05135663) following the successful completion of phase I/II trials. A press release regarding the phase I/II study from NS Pharma identified that no serious adverse effects occurred during the trial which required discontinuation (NCNP, 2022).

**DS-5141B:** In the realm of exon skipping therapy, another noteworthy chemically modified nucleic acid is the 2′-O, 4′-C-ethylene-bridged nucleic acid (ENA). Leveraging this chemistry, Daiichi-Sankyo has developed an ASO named DS-5141B, designed to induce dystrophin mRNA exon 45 skipping for the treatment of DMD (Takaishi et al., 2017, Daiichi Sankyo Co., 2023). ENA-based ASOs have demonstrated increased exon skipping efficiency in both skeletal and cardiac tissue in mouse studies compared to PMOs, a critical factor given the high rate of cardiomyopathy-related mortality in DMD(K. Takaishi, 2017). In a phase 1/2 clinical trial, patients with DMD received subcutaneous administration of DS-5141B once weekly for 12 weeks, followed by weekly doses for 48 weeks. Since no safety concerns, such as treatment discontinuation or clinically significant adverse events, were observed, they initiated a phase 2 extension study in 2022 (Daiichi Sankyo Co., 2021). However, the development of DS-5141B was terminated in April 2023 by Daiichi Sankyo (Daiichi Sankyo Co., 2023).

Apart from the exon skipping ASO, there is another ASO in clinical trials that works by an RNase H-mediated mechanism (Woodcock et al., 2024). **ATL1102:** ATL1102 is a second-generation immunomodulatory 2′MOE gapmer antisense oligonucleotide that specifically targets human CD49d RNA. When ATL1102 binds to the CD49d RNA, intracellular RNase H attaches and downregulates the CD49d RNA. Children with Duchenne Muscular Dystrophy (DMD) have dystrophin-deficient muscles, making them susceptible to contraction-induced muscle injury, which triggers the immune system and exacerbates muscle damage. CD49d is a biomarker of disease severity in DMD; higher numbers of CD49d-expressing T cells correlate with more severe and progressive weakness, even with corticosteroid treatment. Phase IIa trials of ATL1102 in Australia showed promising safety results. After 24 weeks, no serious treatment-related adverse effects were observed. Although blood lymphocyte counts remained stable, there was a notable increase in CD3+, CD49d + T lymphocytes four weeks post-treatment cessation. Grip and pinch strength showed no significant changes. Despite the lack of details on further trials, the favorable safety profile and potential stabilizing effect of ATL1102 suggest optimism for its continued development (Woodcock et al., 2024).

## 3 PPMO as a therapeutic approach for DMD

In addition to adverse reactions, currently approved PMO-based therapeutics for DMD are accompanied by several limitations that highlight the challenges of utilizing PMOs as therapeutic agents. One notable limitation is the poor cellular uptake and the limited ability of PMOs to effectively penetrate cellular membranes due to their neutral nature. This impedes their ability to reach and engage with their intended cellular targets, resulting in reduced therapeutic efficacy. Furthermore, PMOs are subject to rapid clearance from the bloodstream, leading to reduced bioavailability and overall effectiveness. This rapid clearance necessitates higher drug doses to achieve the desired therapeutic effects, potentially posing safety and compliance challenges for patients (Lu et al., 2011). Additionally, the variability in dystrophin expression and distribution across different target tissues, and even within the same tissue, introduces complexity to the therapeutic approach. This variability makes it challenging to attain consistent and predictable treatment outcomes. To address these limitations, ongoing research has explored the use of PPMOs (PMOs conjugated to cell-penetrating peptides) (Moulton and Moulton, 2010, Tsoumpra et al., 2019). PPMOs are designed to enhance cellular uptake and improve therapeutic effectiveness, presenting a potential solution to optimize PMO-based therapies and overcome the challenges associated with their application in treating DMD.

## 4 Adverse reaction and toxicity of PPMO in clinical trial/preclinical/phase I clinical trials for DMD

### 4.1 SRP-5051

Sarepta Therapeutics has designed a PPMO, a CPP conjugated to eteplirsen (SRP-5051), targeting exon 51 that is currently in phase II clinical trials for DMD. SRP-5051 targets the same patient population as eteplirsen but incorporates a proprietary CPP, making it a next-generation version of eteplirsen (ClinicalTrials.gov ID: NCT04004065). While the CPP can increase tissue infiltration and cellular uptake of the ASO therapy, there are concerns about increased toxicity due to the charged nature of the CPP. The Phase I study on safety and tolerability (NCT03375255) was completed in August 2019 and a Phase II dose-determination trial is currently being investigated (NCT04004065). Preliminary results suggest monthly 30 mg/kg dose of SRP-5051 has an 18-fold increase in dystrophin exon skipping and an 8-fold increase in dystrophin protein production compared to etepilersen dosed weekly (Sarepta Therapeutics, 2022). However, the clinical trials have reported adverse events related to the study drug, even at a relatively modest dosage of 10 mg/kg. Notably, patients receiving 30 mg/kg experienced seven out of twelve adverse events, including three cases of hypomagnesemia, which led to a temporary halt in the trial. The precise mechanism underlying hypomagnesemia with ASO-peptide conjugates remains uncertain. However, it is plausible to propose a hypothesis suggesting a link between hypomagnesemia and renal impairment. It is conceivable that renal dysfunction may disrupt magnesium reabsorption processes, thereby contributing to hypomagnesemia observed in patients receiving ASO-peptide conjugates. Further research is warranted to elucidate the specific mechanisms underlying this adverse event (Aartsma-Rus et al., 2023). Hypomagnesemia was successfully managed with supplementation, and the FDA lifted the clinical hold in September 2022 (Sarepta Therapeutics, 2022). The ongoing Phase II trials are expected to conclude in August 2024, providing further insights into the safety and preliminary efficacy of dystrophin restoration with SRP-5051. The occurrence of hypomagnesemia, not associated with eteplirsen treatment, raises concerns about potential adverse effects related to the CPP component of SRP-5051. According to the latest press release in January 2024, findings from the MOMENTUM study reveal that SRP-5051 at a dosage of 30 mg/kg led to a significant 24.6-fold increase in exon skipping, resulting in a 12.2-fold increase in dystrophin expression compared to the same dose of eteplirsen after 24 weeks (NCT04004065). Additionally, similar observations were made with the lower 20 mg/kg dose, where severe hypomagnesia and hypokalemia occurred in 4 and 3 patients, respectively. However, it is noteworthy that these adverse reactions did not result in any treatment discontinuations (Meglio, 2024b).

### 4.2 PGN-ED051

PepGen has designed an enhanced delivery oligonucleotide (EDO) platform that is conjugated to an ASO aiming to skip DMD exon 51 for the treatment of DMD (INC, 2022). The EDO consists of Pip CPPs that have been optimized for cell infiltration and safety. These Pip peptides are characterized by their high content of arginine and aminohexanoic acid residues (Klein et al., 2019). PGN-ED051 has just completed the Phase 1 trial with healthy normal volunteers, demonstrating the safety of the drug at 1, 5, 10, or 15 mg/kg dosages (INC, 2022). No adverse events were reported at the 1 and 5 mg/kg doses. At the 10 mg/kg dose there was only 1 mild adverse event reported. At 15 mg/kg doses, there were problems in the kidneys which was reported as a non-life-threatening serious adverse event. Additionally, mild to moderate hypomagnesemia was observed in two participants (INC, 2022). This along with the same adverse event reported for SRP-5051 suggests this adverse event may be due to the actions of the CPP in general. A Phase 2 trial to evaluate the safety and tolerability of ascending doses in DMD patients started in October 2023 (NCT06079736).

### 4.3 ENTR-601-44

Entrada Therapeutics has developed ENTR-601-44, a Peptide conjugated PMO (PPMO) designed to restore functional dystrophin production through exon 44 skipping(Entrada Therapeutics, 2022). This PPMO utilizes Endosomal Escape Vehicle (EEV) platform developed by Entrada, which induces the collapse of endosomes containing the PPMO, enhancing its bioavailability and effectiveness(Qian et al., 2016, Sahni et al., 2020). The company is currently in the process of submitting an Investigational New Drug (IND) application to the FDA for a phase 1 clinical trial of ENTR-601-44. However, a clinical hold imposed by the FDA in December 2022 may cause a delay in this process(Entrada Therapeutics, 2022). As of November 2023, the FDA has not lifted the clinical hold, despite Entrada Therapeutics providing additional information to address their concerns. However, Entrada Therapeutics has received approval from regulatory authorities in the United Kingdom to conduct a phase 1 clinical trial of ENTR-601-44. This UK-based trial is set to involve healthy volunteers and will assess the safety, tolerability, pharmacokinetics, and target engagement of the drug (Waldron, 2023). Currently, the first and second cohorts have completed their dosings for this drug and further details, including information on therapy and toxicity, are anticipated to become available in 2024 (Entrada Therapeutics, 2023).

### 4.4 Other approaches to improve ASO delivery

Apart from cell penetrating peptides, antibody conjugation with PMOs presents a promising avenue for enhancing the targeted delivery of oligonucleotides to muscle tissues. This approach involves utilizing antibodies that target receptors highly expressed on the muscle cell membrane, facilitating direct internalization of oligonucleotides into muscle cells. Recent advancements in this field have been marked by the introduction of antibody-conjugated AONS hybrids by both Dyne Therapeutics and Avidity Biosciences. These hybrids, paired with transferrin receptor 1 (TfR1) antibodies, represent a novel approach to targeted oligonucleotide delivery. The choice of TfR1 is significant, given its ubiquitous presence on the cell membranes of skeletal, smooth, and cardiac muscles, as well as its expression in the brain (Filonova and Aartsma-Rus, 2023, Wilton-Clark and Yokota, 2023).

**DYNE-251:** DYNE-251 is an exon skipping therapy utilizing an antigen-binding fragment (Fab) to facilitate the delivery of antisense oligonucleotide to muscle cells, particularly for individuals amenable to exon 51 skipping. Currently, the first clinical trial of DYNE-251 is underway, aiming to evaluate its safety, dystrophin restoration, and clinical efficacy over 24 and 120 weeks of treatment. The trial is anticipated to conclude in November 2026. Newly announced initial data from the phase 1/2 DELIVER trial (NCT05524883) demonstrated that treatment with DYNE-251 showed 0.88 % mean dystrophin expression at 6 Months in 5 mg/kg Cohort Administered Monthly which is at least a 2.5-fold higher than the eteplirsen study. Patients treated with 10 mg/kg of DYNE-251 Q4W had a mean dystrophin level of 3.22 % of normal and a 2.97 % change from baseline at 6 months. When adjusted for muscle content, the mean absolute dystrophin level reached 7.64 %, which is higher than levels reported by other peptide conjugate PMOs in development (Dyne Therapeutics, 2024). Safety evaluations, involving 45 patients enrolled in the 5.4 mg/kg Q8W cohort of the MAD phase, confirmed favorable safety profile of DYNE-251. The most common treatment-related adverse events, such as headache (16 %), nasopharyngitis (16 %), vomiting (14 %), infusion-related reactions (11 %), falls (11 %), and cough (11 %), were generally mild or moderate in severity. Only one serious adverse event, dehydration due to gastroenteritis, was observed, which was not attributed to the study drug (Filonova and Aartsma-Rus, 2023, Wilton-Clark and Yokota, 2023, Therapeutics, 2024).

**AOC 1044:** AOC 1044, developed by Avidity, is an exon skipping therapy tailored to target exon 44 of the dystrophin gene in individuals with DMD mutations amenable to this skipping. It utilizes a monoclonal antibody (mAb) specifically targeting TfR1 to deliver PMOs to skeletal muscle and heart tissue. In January, Avidity released a press statement detailing the ongoing assessment of AOC 1044 in EXPLORE44, a phase 1 trial comprising both healthy volunteers and patients with DMD. Recently unveiled topline data from this trial, involving 40 healthy volunteers, revealed remarkable dose-dependent enhancements in PMO concentrations within skeletal muscle following a single dose of either 5 mg/kg or 10 mg/kg of AOC 1044. These increases were notably higher compared to those achieved with a single dose of conjugated PMOs. Moreover, at day 29, patients administered a single dose of 10 mg AOC 1044 exhibited statistically significant exon 44 skipping of up to 1.5 % when compared to the placebo group. At the time of writing no information regarding the safety was available (Filonova and Aartsma-Rus, 2023, Wilton-Clark and Yokota, 2023, Meglio, 2024a).

## 5 ASO based therapy compared to other treatment approaches for DMD:

ASO-based therapy has shown promising results including increased dystrophin production, improved muscle function, and slow disease progression in preclinical and clinical studies, with four ASOs approved for the treatment of DMD and several in clinical trials. Most importantly ASOs have demonstrated a favorable safety profile in clinical trials, with most adverse events being mild to moderate and transient (Duan et al., 2021). Apart from that The FDA has recently granted approval to a gene therapy, delandistrogene moxeparvovec (Elevidys), by Sarepta for Duchenne muscular dystrophy (DMD) (Mullard, 2023). This single-dose gene transfer therapy utilizes a recombinant adeno-associated virus vector serotype 74 (AAV74) to deliver a microdystrophin transgene encoding a dystrophin protein (Shoti et al., 2023). Sarepta chose AAV74 over AAV9 due to its potential for better safety and efficacy profiles. Previous clinical trials using AAV9 vectors reported several adverse events. In a Phase I/II trial by Solid Biosciences, adverse events such as complement activation and thrombocytopenia leading to kidney injury and cardiopulmonary insufficiency were observed, despite more recent data showing clinical improvements. In a Pfizer-sponsored trial, serious adverse events including acute kidney injury, atypical hemolytic uremic syndrome, thrombocytopenia, and the death of a patient at a high dose were reported. Additionally, a patient died in an N-of-1 clinical trial using AAV9-CRISPR sponsored by Cure Rare Disease (Shoti et al., 2023).

In contrast, AAV74 has shown only manageable adverse events, such as nausea and vomiting, in all participants. Preliminary results from clinical trials using different versions of micro-dystrophin cDNA with various AAV serotypes are promising. However, there are concerns about the longevity of the micro-dystrophin transgene due to the rare integration of AAVs into the host genome, potentially leading to transgene loss over time with muscle turnover (DUAN, 2018a, Duan et al., 2021). Additionally, AAV administration may induce anti-AAV capsid neutralizing antibodies, preventing retreatment (Duan et al., 2021). Gene therapy approaches also carry potential risks of insertional mutagenesis and off-target effects (Wilton-Clark and Yokota, 2023).

Glucocorticoids, the current standard of care for DMD, have shown efficacy in delaying disease progression and prolonging ambulation. However, their non-selective action contributes to numerous complications, prompting exploration of alternative steroids like vamorolone, which are being investigated for their improved safety profile. While initial trials indicate tolerability, ongoing placebo-controlled trials are essential to ascertain efficacy (Heier et al., 2013, Reeves et al., 2013, Hoffman et al., 2019).

Other treatment approaches targeting secondary consequences of dystrophin loss include myostatin inhibition and mitochondrial function improvement. However, these approaches have shown mixed results and challenges in long-term use and therapeutic efficacy. For example, while myostatin inhibition has demonstrated improvement in muscle mass in healthy subjects, adverse effects have hindered its long-term use in DMD patients (Duan et al., 2021). Givinostat, a Histone deacetylase inhibitior, has shown reduced fibrosis in a small study(Mullard, 2024). Besides, when compared to placebo, Givinostat met its primary endpoint with secondary and exploratory endpoints showing consistency with the primary endpoint, prompting the FDA approval for DMD patients (Mercuri et al., 2024).

In summary, while ASO-based therapy offers a targeted and safe treatment approach for DMD, it is essential to recognize the strengths and limitations of alternative treatment modalities. Continued research and development are crucial to address the unmet needs of DMD patients and improve their quality of life.

## 6 Conclusion and future directions

Antisense Oligonucleotide-based drugs offer great potential for treating genetic diseases like Duchenne muscular dystrophy (DMD), but their limited distribution in the body has posed challenges. To overcome this, cell-penetrating peptides have been explored to improve drug delivery. However, the adverse reaction and toxicity of both ASO and peptide-conjugated ASO drugs is a critical concern that must be carefully considered to ensure better drug safety.

This review has provided an overview of adverse reaction and toxicity data for four FDA-approved ASO drugs for DMD, as well as peptide-conjugated ASOs in preclinical studies and clinical trials. One notable limitation of existing safety data is the small number of patients included in clinical trials, which is understandable due to the rare nature of this disease. Nevertheless, both common and less common adverse drug reactions and severe toxicity have been observed within this drug class. It is worth noting that while some adverse reactions have been documented in clinical studies, these findings may not fully represent real-world scenarios where ASO drugs are used to address diseases on a global scale. Therefore, it is imperative to extend observations from clinical trials to real-world postmarketing settings to validate findings on ADRs and toxicity.

A significant challenge in the field of ASO drugs is understanding the mechanisms behind hepatotoxicity, renal toxicity, and hypersensitivity reactions. While there are speculations about these mechanisms, the precise genetic-level events remain unclear. Generally, as PMO is neutral in charge, they do not readily interact with proteins compared with other charged ASOs (Nan and Zhang, 2018). Therefore, this suggests that they will have minimal hybridization-independent protein interactions leading to increased immunogenic responses. Mouse studies have indicated an increased immune response to the new truncated dystrophin produced from the exon-skipping ability of the PMO treatment (Vila et al., 2015). Therefore, the newly produced truncated dystrophin as a result of the PMO increasing exon-skipping may be inducing an immunogenic response. Additionally, data on kidney dysfunction primarily come from preclinical studies, with limited evidence from clinical trials and post-marketing surveillance due to the small number of patients treated with ASO drugs. With the ongoing development and subsequent approval of more ASO and peptide-conjugated ASO drugs for the treatment of diseases like DMD, there is an urgent need to gain a better understanding of the risks associated with this class of drugs. Expanding the patient population and conducting comprehensive studies will be crucial in achieving this goal.

Lastly, in light of the rapid progress in ASO drug development, there is an immediate need to tackle critical inquiries. Two pivotal questions include: 1) What are the fundamental molecular mechanisms responsible for ASO drug-induced toxicity? and 2) How can pharmacological strategies be employed to mitigate the risks linked to ASO drug-induced toxicity? Addressing these questions has the potential to pave the way for various promising future directions, ultimately contributing to the success and safety of ASO and peptide-conjugated ASO-based drugs. This progress can extend beyond the treatment of Duchenne muscular dystrophy (DMD) to address a multitude of devastating genetic diseases.

## CRediT authorship contribution statement

**Umme Sabrina Haque:** Conceptualization, Methodology, Writing – original draft. **Melissa Kohut:** Writing – review & editing. **Toshifumi Yokota:** Conceptualization, Methodology, Supervision, Writing – review & editing.

## Funding

This research received no external funding.

## Declaration of competing interest

The authors declare the following financial interests/personal relationships which may be considered as potential competing interests: T.Y. is a co-founder and shareholder of OligomicsTx Inc., which aims to commercialize antisense technology. The remaining author declares that the research was conducted in the absence of any commercial or financial relationships that could be construed as potential conflicts of interest.

## Data Availability

No data was used for the research described in the article.
